# Saunders’s Medical Hand-Atlases

**Published:** 1902-07

**Authors:** 


Saunders's Medical Hand-Atlases. Atlas and Epitomé of Otology. By Gustave Bruhl, M. D., of Berlin, with the collaboration of Professor Dr. A. Politzer of Vienna. Edited, with additions, by S. MacCuen Smith, M. D., Clinical Professor of Otology, Jefferson Medical College, Philadelphia. With 244 colored figures on 39 lithographic plates, 99 text illustrations and 292 pages of text. Philadelphia and London : W. B. Saunders & Co. 1902. [Cloth, $3.00 net.]

With all the credit due to Saunders for the excellent manner in which this book has been published, and that due Brühl, the author, and Prof. Politzer, the collaborator, the American editor, Dr. S. MacCuen Smith, of Philadelphia, must not be overlooked. It is largely due to his excellent and common-sense handling of the work that it is as valuable as it really is, as a text-book and a teacher of a specialty. The book is a splendid additon to the Saunders series, and aims to be both didactic and clinical in its teaching. It is a work which will be of untold benefit to students and of rare assistance to the general practitioner, who every day in his practice runs across ear cases which he will find clearly set forth, described, pictured and treated in this otology.

The illustrations comprise 244 colored figures and 99 text illustrations, showing the anatomy of the ear and portraying pathologic changes in so excellent a manner as to make them of untold value.

Dr. Brühl was extremely fortunate in having his former chief, Prof. Politzer, in collaboration on the work, for in addition to the latter's advice and deep scientific knowledge, his rare collection of specimens was freely used in illustrating, and have done much to make the volume the very complete work it is.

